# Emergency contraception in primary health care

**DOI:** 10.4102/safp.v67i1.6146

**Published:** 2025-06-17

**Authors:** Indiran Govender, Olukayode A. Adeleke, Lusayn L. Govender, Olufemi B. Omole

**Affiliations:** 1 Department of Family Medicine and Primary Health Care, Walter Sisulu University, Mthatha, South Africa; 2 Department of Family Medicine and Rural Health, Faculty of Medicine and Health Sciences, Walter Sisulu University, Mthatha, South Africa; 3 Department of Occupational Therapy, Charles Sturt University, Bathurst, Australia; 4 Department of Family Medicine and Primary Care, Faculty of Health Sciences, University of the Witwatersrand, Johannesburg, South Africa

**Keywords:** unwanted pregnancies, unplanned pregnancies, economic stress, emergency contraception, intra uterine contraceptive device, Copper IUD, unprotected sexual intercourse, rape, sexual assault

## Abstract

Unplanned and unwanted pregnancies in South Africa cost women, government and families enormous physical, emotional and socioeconomic stress. These are further aggravated by the high rate of sexual assault cases in South Africa. In a setting such as this, easy access to emergency contraception (EC) becomes a key intervention and health system imperative. Primary health care clinicians are at the forefront of health care provision in South Africa and need to be well equipped with the necessary knowledge on EC to make informed management decisions. This article seeks to provide information and improve awareness and confidence of primary care clinicians when providing EC.

## Introduction

Emergency contraception (EC) is important for the South African population because of the high number of unplanned and unwanted pregnancies. There are more than 14 million cases of unplanned pregnancies recorded per year.^1,2,3^ Emergency contraception involves various methods used to prevent pregnancy after unprotected sexual intercourse,^1^ sexual assault^1^ or contraceptive failure.^2^ Emergency contraception, unlike regular contraception, is used after sexual intercourse but before the potential time of implantation.^1^ Emergency contraception use should not replace the regular contraception, and regular contraception methods should be offered following the use of EC.

Unprotected sexual intercourse is common, and although there are no accurate data from South Africa, about 14% of women who seek EC in the United States (US) report at least one episode of unprotected intercourse in the previous six days, and up to 40% reported multiple episodes of unprotected intercourse in the cycle before presentation.^2^ Similarly, up to 19% of women in the US report a history of rape or attempted rape,^2^ and as many as 45% of all pregnancies in the US are unplanned.^4^ In South Africa, rape is also a major problem, with the country ranking first globally with a per capita rate of 417 out of 100 000 women reporting rape.^5,6^ This high rate of sexual assault is one of the driving indications for EC use in South Africa. In a recent study conducted among human immunodeficiency virus (HIV)-positive women in Eastern Cape province, up to 71% of pregnancies were unplanned.^7^ Despite the high rates of unplanned pregnancy and sexual assault among women in South Africa, the sub-Saharan African region, including South Africa, has the lowest rate (29.4%) of contraceptive use globally.^1^ Data on the use of EC in South Africa are very sparse. A scoping review reported that only 11.3% of participants in South African studies reported the use of EC previously. Although the awareness of EC is on the rise in South Africa,^1^ many women, pharmacists, and doctors are not well informed about the available options of EC.^4^ Both the Practical Approach to Care Kit guide (PACK) (used in the Western Cape province), and the Advanced Practice Clinical guidelines (APC) (used outside the Western Cape province) have some pages dedicated to EC in the contraception and sexual assault pages. However, there still appears to be a lack of awareness among medical practitioners. This lack of awareness may partly explain the high numbers of unwanted pregnancies and possibly contribute to the high termination of pregnancy rate and maternal deaths in South Africa.^4^ As EC does not prevent sexually transmitted infections (STIs) including HIV, it is important to also offer HIV prophylaxis and treatment for STI to women seeking EC after sexual exposure or assault.^2^

A study in Johannesburg, South Africa, concluded that despite the country’s commitment to achieving universal access to sexual and reproductive health services, there are challenges with the current contraception services delivery, specifically regarding contraceptive counselling.^8^ In the primary care setting, health care providers play a vital role in offering timely access to EC, including educating patients and addressing any concerns. Health care providers in primary care settings should, therefore, be knowledgeable about the various types of EC and be able to facilitate the provision of the best option for each patient in the correct time frame, in an understanding and sensitive manner.^2^ This article provides up-to-date evidence on the indications, effectiveness and special considerations on commonly used EC methods to health care providers. This may assist in improving access to EC, reduce the number of unplanned pregnancies and potential adverse pregnancy-related outcomes, including maternal deaths.

## Indications for emergency contraception

Emergency contraception is usually indicated for unprotected intercourse,^4^ contraceptive failure (e.g. condom breakage, missed birth control pills),^4^ post sexual assault^9^ and sexual coercion.^4^ In this article, the authors refer to sexual assault as an act of sexual abuse in which one intentionally sexually touches another person without that person’s consent, or physically forces a person to engage in a sex against their will. It is a form of sexual violence.^5,6^ It must be provided to women, including children from the Breast Tanner Stage III.^10^

The effectiveness of EC is a function of the time elapsed from the time of exposure to unprotected sex to the time of administering the EC.^7^ It is, therefore, important that patients at risk of unwanted pregnancy present as early as possible after exposure to unprotected sex. In addition, the clinician needs to observe the time elapsed because it has implications for the choice of the EC method and the ability to effectively prevent unwanted pregnancy.

## Types of emergency contraception methods

Emergency contraception is available either as oral pills or as intrauterine devices (IUDs) (hormonal or non-hormonal). The exact mechanisms of action for ECs are generally unclear but, broadly speaking, may be attributable to interference with follicle maturation or the ovulatory process, cervical mucus, sperm migration, corpus luteum sufficiency, endometrial reproductivity, fertilisation, or zygote development, transport, or adhesion. The choice of EC differs according to the type of EC as well as the time elapsed from exposure to unprotected intercourse and ovulation.^3,11^

### Emergency oral contraceptive pills

**FIGURE 1:**
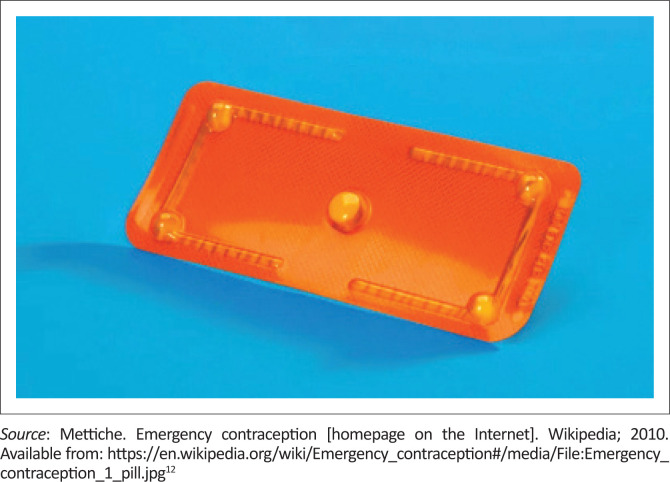
Levonorgestrel as sold in South Africa.

**FIGURE 2:**
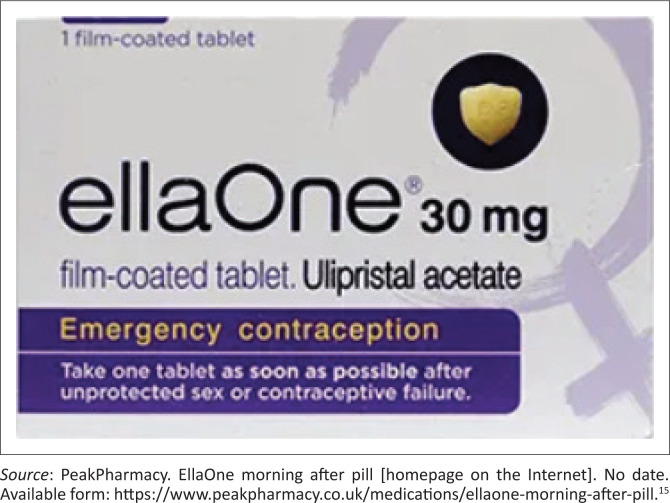
Ulipristal Acetate (UPA) as sold.

The oral contraceptive pills group offers two options, namely: (1) Oral Levonorgestrel (LNG) (see Figure 1^12^); and (2) Ulipristal Acetate (UPA) (see Figure 2^15^).

Oral Levonorgestrel is known for its:

- Over-the-counter availability.^13^
- Mechanism of action involving the inhibition of ovulation.^4^
- Effectiveness within 72 h (three days) after unprotected sex, yet can still be taken up to five days.^4,14^
- Single dose of 1.5 mg orally STAT.^4^
- Lower effectiveness in women with body mass index (BMI) > 30 kg/m^2^.^4^
- Preferred double dose for women with weight > 80 kg or BMI > 30 kg/m^2^ should be advised to double the dose of LNG.^10^

The Ulipristal Acetate (UPA) (Figure 2^15^) is known:

- As a synthetic progesterone receptor modulator that binds progesterone receptor, inhibiting or delaying ovulation.^7^
- For not affecting cervical mucus or sperm motility but inhibits ciliary beating.
- For muscle contraction in the fallopian tubes.^16^ Although animal studies suggest inhibition of implantation, there is insufficient evidence this occurs in humans.^16^
- It is also said to reduce endometrial thickness and delay endometrial maturation, potentially inhibiting implantation.
- Generally, current evidence suggests little post-ovulatory action for commonly used hormonal contraceptives, and no action on foetal development and no abortifacient effect.^17,18^
- Available only on prescription^13^ but currently not in South Africa.
- Superior efficacy in obese women compared to LNG.^13^
- Maintains higher efficacy throughout the five day window compared to LNG.^14^
- Effective up to 120 h; (five days) after unprotected sex.
- Single 30 mg dose orally.^4^

The combined oral contraceptives (Yuzpe’s method or regimen)^4^:

- The least effective method of oral EC, with the most side effects, particularly nausea and vomiting.^4,19^
- Contain 100 µg ethinylestradiol and 1 mg norgestrel (e.g. two Ovral^®^ tablets, i.e. combined oral contraceptive tablets) taken 12 h apart.^4,20^
- Two Ovral^®^ tablets are taken orally stat and repeated 12 h later.^4^
- Must be taken within 72 h of unprotected intercourse.^4^
- Available as other trade names but must contain the required dose of ethinylestradiol and norgestrel to be effective.^4^

### Intrauterine devices

These devices are very effective and strongly recommended by the World Health Organization (WHO) for EC.^21^ There are two commonly used types – copper and hormonal IUDs. They prevent fertilisation by altering the endometrium and fallopian tube cell linings to induce an environment hostile to the sperm and even the fertilised egg. The inflammatory response that is induced by IUDs and the increased levels of leukocytes and prostaglandins in the fallopian tubes also create an environment that is detrimental to both sperm and egg, further reducing the likelihood of fertilisation.^22^

**FIGURE 3:**
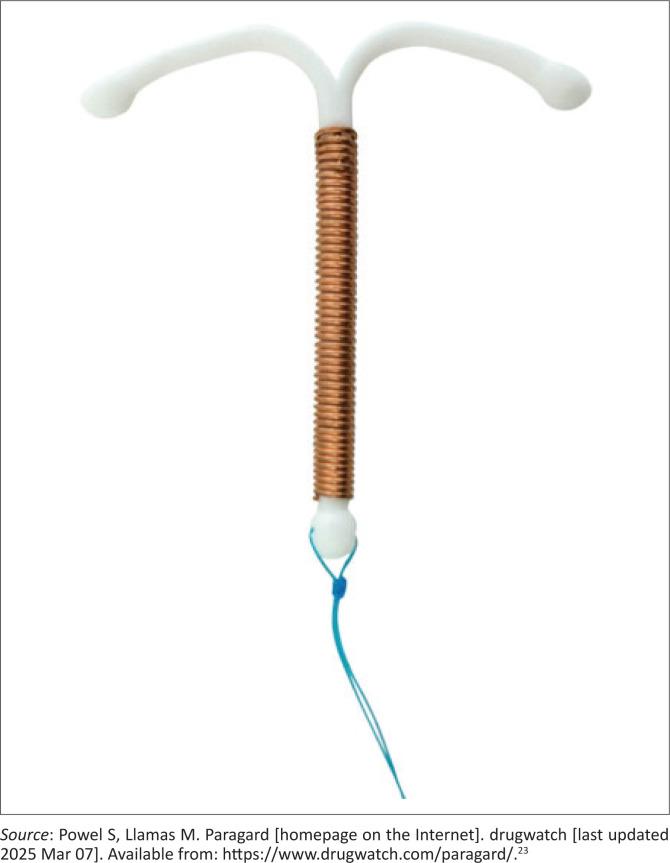
Copper intrauterine device.

The Copper intrauterine device or ‘Copper T’ (Figure 3^23^) is known for:

- The presence of copper ions released by the device increases the levels of copper in the uterine and tubal fluids. Copper is toxic to sperm, reducing their motility and viability. This prevents the sperm from fertilising the egg.^21,22^ It induces an inflammatory reaction in the endometrium, creating a hostile environment for any fertilised egg and preventing implantation.^4^
- Most effective of all EC methods.^4^
- It requires insertion by a health care professional.
- Can be used as EC if inserted within 120 h (5 days) of unprotected sex.
- In addition, it provides ongoing contraception from the time of insertion for up to 10 years.^4,14,24^

**FIGURE 4:**
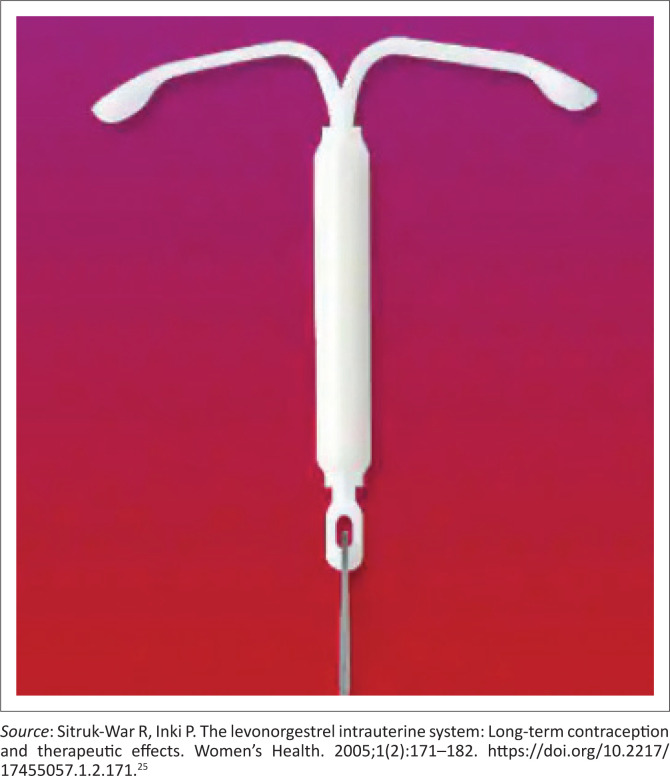
Levonorgestrel intrauterine device.

The Levonorgestrel (LNG) intrauterine device (Figure 4^25^) is characterised by:

- This device is less commonly used for EC compared to copper IUDs (possibly because of the higher cost and expertise required). It releases a hormone called levonorgestrel, which thickens the cervical mucus, inhibits sperm motility and function and alters the endometrial lining to prevent fertilisation and implantation.^22,26^
- It is commonly known as ‘Mirena’.
- It is equally effective as copper IUD for EC (Turok et al 2021).^27^
- Its efficacy is not affected by body mass but it is it is costlier than Copper T.^27^
- Can be used as EC if inserted within 120 h (five days) of unprotected sex.^27^

### Important considerations about the use of different emergency contraception methods

Table 1 shows a comparison of the different ECs based on the considerations when choosing an appropriate EC. The effectiveness of the various ECs is influenced by several considerations, including:

**TABLE 1:** A comparison of the different emergency contraceptions.

Type of contraception	Efficacy	Relative cost	Accessibility	Skills required for use	Optimal time (hours)
Oral LNG	=> *80% effective*	Cheap	Public health care and Private health care	None	72 yet usable up 120
Oral ulipristal	Effective+	Cheap	Not available in South Africa	None	120
Combined oral pill (Yuzpe method)	Least effective of the oral pills	Cheap	Public health care and private health care	None	72
Copper T IUD or ‘Copper T’	=> *90% effective*	Expensive	Public health care (free) Private health care (expensive)	Insertion skills	120
Hormonal IUD (Mirena)	Very effective	Expensive	Public health care (free) Private health care (expensive)	Insertion skills	120

LNG, Levonorgestrel; IUD, intrauterine devices.

#### Availability

Over-the-counter EC may be the most preferred because of the perceived privacy, comfort, convenience and efficacy. Concerns were raised that the readily available drugs may increase risky sexual behaviour, but a systematic review showed that this is not the case.^28^

#### Timing of use

According to the South African National Contraception Clinical Guidelines (2019) and Atkins et al, except for oral EC pills, others are effective within 120 h (5 days) after unprotected sex. The earlier it is used, the more effective it is.^29^

#### Repeated unprotected sexual intercourse in the same cycle

Data suggest that repeated unprotected sex during the same menstrual cycle may be associated with increased risk of EC failure. So, considering that EC oral pills delay ovulation, abstinence from sex or the use of additional protection is needed with their use as EC.^19^

#### Body weight

Levonorgestrel may be less effective in individuals with a higher body mass index (BMI), and in such instance, ulipristal acetate or the copper IUD is preferred.^4^

#### Breastfeeding

Levonorgestrel and IUDs are safe during breastfeeding but the use of ulipristal acetate may require temporary cessation of breastfeeding.^2,4^

#### Drug interactions

Some medications (These are cytochrome p450 (CYP450) enzyme inducers that may decrease the efficacy of the emergency hormonal contraception; drugs include carbamazepine, rifampicin, phenytoin and griseofulvin.) can reduce the effectiveness of EC pills, and health care providers should review the patient’s medication history.^10,30,31^ In situations where vomiting occurs within 3 h of ingesting EC oral pills, a full redosing is warranted. Co-administration with an antiemetic such as metoclopramide may be beneficial.

#### Sexually transmitted infections

Some studies suggest that unrestricted availability of EC may increase risky sexual behaviour, less condom usage and a higher risk of STIs.^32^ However, this increased risk is not consistently found for all types of STI.^33^

#### Frequent use of emergency contraception

This should signal a strong need for long-term contraception, and the health care provider should use the opportunity of the visit to counsel the patient as such.

#### Barriers to access

**Cost:** The cost of EC varies, depending on the pharmacy and the type of EC. This may pose a challenge for patients who do not have private medical insurance or undocumented immigrants, who are not eligible for free public health care services in South Africa. Health care providers in private practice need to be aware of free or low-cost EC options. However, EC is offered at no cost in the public primary health care clinics and these clinics are open from 08:00 to 16:00 on weekdays,^34^ and some are open twenty-four seven (24/7). Although the IUDs are the most effective, they are also the costliest at the time of insertion. The requirement for insertion skills creates additional costs, particularly that more specialised health care providers may render higher service fees for inserting the IUDs. It should, however, be noticed that their contraceptive effects may last up to 10 years after insertion, and this makes it cheaper than other EC methods in the long term.^4,14,24^

**Awareness and knowledge:** Patients may not know about EC options or how to access them. Community education and engaging in opportunistic prevention during clinic visits are means of increasing awareness. Pharmacists may also play a pivotal role in creating awareness regarding the availability and effectiveness of EC during over-the-counter consultations.^2^

**Cultural and religious beliefs:** These may include deeply rooted myths and negatively affect patient’s willingness to use EC, dictating that health care providers not only provide health education to clients to demystify EC but also be culturally sensitive while engaging them.^29,32^

**Health care provider skills:** Although IUDs are freely available in the public health care services as part of the long-acting reversible regular contraception, the unavailability of a health care provider skilled in its insertion may negate its uptake at the time a woman seeks EC.^2^ Additional charges for insertion by the health care provider in private practice may also debar uptake.^35^

## Contraindications to emergency contraception

### Pregnancy

This is the only absolute contraindication to EC. Nonetheless, no adverse effects have been reported following the inadvertent use of EC pills or ulipristal in early pregnancy, as these are not abortifacients or cause foetal malformations.^19^

### Intrauterine devices

These devices should not be used in uterine abnormalities, current pregnancy, abnormal uterine bleeding, Wilson’s disease (Copper T), gynaecological malignancies, pelvic inflammatory disease or pelvic tuberculosis.^4^

### Hypercoagulable states

Oral EC pills should not be taken.^4^ These states include antiphospholipid syndrome, antithrombin III deficiency, prothrombin 20210 mutation, factor V Leiden, lupus anticoagulant, protein C deficiency and protein S deficiency.

## Conclusion

Ensuring good pregnancy outcomes requires a woman to conceive when her health and socioeconomic conditions are optimal. The high rates of unplanned pregnancy and sexual assault, and the poor outcomes of maternal and women’s health care services in South Africa, all necessitate promoting the use of EC. Health care providers in primary health care (PHC) are best placed to ensure that women are educated on EC and have timely access to this important service. While the information provided in this article has potential to scale up the use of EC and promote good reproductive health outcomes, training of health care providers and uninterrupted supply of different types of ECs are other key imperatives.
